# Effects of Serine Protease Inhibitors on Growth and Development and Digestive Serine Proteinases of the Sunn Pest, *Eurygaster integriceps*

**DOI:** 10.1673/031.011.7201

**Published:** 2011-05-29

**Authors:** Fatemeh Saadati, Ali R. Bandani

**Affiliations:** Plant Protection Department, College of Agriculture and Natural Resources, University of Tehran, Karaj, Iran

**Keywords:** adult weight, digestive proteinase, inhibitors, nymphal development time, survival

## Abstract

In the current study the effects of serine proteinase inhibitors (TLCK, TPCK, SBTI, and a combination of SBTI and TPCK) with concentrations of 1% and 4% of dietary protein in artificial diets were tested against growth of the Sunn pest, *Eurygaster integriceps* Puton (Hemiptera: Scutelleridae), development, and its gut serine proteinase targets. Analysis of variance indicated that protease inhibitors affected nymphal development time, adult weight, and survival. Mean development time of third instar nymphs in control, SBTI (1%), TLCK (1%), and TPCK was 7.18, 9.74, 9.97, and 8.52 days, respectively. The highest mortality (100 % mortality) was observed when a combination of TPCK and SBTI, both at 4% of dietary protein, was used followed by TPCK (4%) that produced 95% mortality. There were significant differences in proteinase activity between treatments and controls when BApNA and SAAPFpNA were used as substrates for trypsin and chymotrypsin, respectively. Reduction of trypsin activity in insects fed with low doses of SBTI (1%), TLCK (1%), and both doses of TPCK (1% and 4%) was 40, 26, 23, and 17%, respectively. Inhibition of chymotrypsin activity was seen in the insects fed on SBTI (1%), TLCK (1%), and TPCK (4%) where inhibition was 14, 9, and 36%, respectively. Maximum inhibition of chymotrypsin activity was observed in the insects fed on diets containing high doses of TPCK (4%). In gel assays, the greatest effects were observed when *E. integriceps* were fed on high doses of SBTI and TPCK. Therefore, TPCK followed by SBTI proved to be the most effective proteinase inhibitors of *E. integriceps*.

## Introduction

Sunn pest, *Eurygaster integriceps* Puton (Hemiptera: Scutelleridae), is a serious pest of cereals in the wide area of the globe from Near and Middle East to East and South Europe and North Africa (Critchley 1998). *E. integriceps* causes severe quantitative and qualitative damage to crops (sometimes up to 100%) by feeding on leaves, stems, and grains. Feeding on grain is the most destructive. *E. integriceps* sucks nutrients from the grain by piercing it with their mouthparts and injecting their salivary enzymes, which contain amylase and proteases (Bandani et al. 2009; Hosseini-Naveh et al. 2009). Salivary secretions of Hemipterans contain a full complement of digestive enzymes for food digestion (Miles 1972; Laurema et al. 1985). By injecting salivary enzymes into the grain during feeding, enzymes degrade gluten proteins, which are divided into two groups: the monomeric gliadins and the polymeric glutenins, with the latter being further classified into high and low molecular weight subunits (Tosi et al. 2009). Pesticide spraying is the main method for *E. integriceps* control in areas where infestation is high. In addition to the high cost of chemical control, insecticides pose a risk to nature's balance, human health, water quality, wildlife, and the environment as a whole. Thus a search for new control methods is needed to diminish reliance on insecticides for insect control. Genetic manipulation of plants offer alternatives to synthetic pesticides by creating insect-resistant plants (Ryan 1990). Plants synthesize a wide range of molecules such as proteinase inhibitors, α-amylase inhibitors, lectins, and chitin binding proteins to resist herbivore insects, pathogens, and wounding (Gatehouse and Gatehouse 1998; De Leo et al. 2001; Silva et al. 2006). Among these proteins, plant protease inhibitors constitute major tools for improving the resistance of plants to insects. Protease inhibitors are tested against insect pests using both in *in vitro* assays using gut proteases and in *in vivo* assays using artificial diet bioassays (Lawrence and Koundal 2002). Proteinase inhibitors are capable of interfering with insect protein digestion by binding to digestive proteases of phytophagous insects, resulting in an amino acid deficiency thus affecting insect growth and development, fecundity, and survival (Lawrence and Koundal 2002; Oppert et al. 2003; Azzouz et al. 2005). Transgenic plants expressing serine and systeine proteinase inhibitors have shown some resistance to Lepidoptera and Coleoptera (De Leo et al. 2001; Falco and Silva-Filho 2003; Alfonso-Rubi et al. 2003). Proteinase inhibitors are the products of single genes, therefore they have practical advantages over genes encoding for complex pathways and they are effective against a wide range of insect pests, i.e. transferring trypsin inhibitor gene from *Vigna unguiculata* to tobacco conferred resistance against lepidopteran insect species such as *Heliothis* and *Spodoptera*, and coleopteran species such as *Diabrotica* and *Anthonomus* (Hilder et al. 1987). It has already been found that *E. integriceps* salivary glands secretions contain mostly serine protease activities, e.g. trypsinand chymotrypsin-like activities (Hosseini-Naveh et al. 2009).

No studies have been done to evaluate the effects of protease inhibitors on *E. integriceps*. Thus, aim of the current study was to investigate the effect of trypsin inhibitor (TLCK, tosyl-L-lysine chloromethyl ketone), chymotrypsin inhibitor (TPCK, N-tosyl-L-phenylalanine chloromethyl Ketone), soybean trypsin inhibitor (SBTi), and combination of SBTI and TPCK in artificial diet against *E. integriceps* growth, development, and its gut serine proteinase targets.

## Materials and Methods

### Substrates and inhibitors

The enzyme substrates BApNA (Na-benzoyl-L-arginine p-nitroanilide), SAAPFpNA (N-succinyl-alanine-alanine-proline-phenylalanin p-nitroanilidine), Azocasein and inhibitors TLCK (Na-p-tosyl-L-lysine chloromethyl ketone), TPCK (Na-p-tosyl-L-phenylalane chloromethyl ketone), and soybean trypsin inhibitors (SBTI) were purchased from Sigma (www.sigmaaldrich.com). Preparation of substrates and inhibitors were done according to manufacturer's instruction.

### Insect culture

The insects were collected from the wheat farm during spring when feeding started. They were fed and maintained on wheat grains in laboratory conditions at 25 ± 2° C and a photoperiod of 14:10 (L:D) (Allahyari M, personal communication).

### Diet preparation and insect bioassay

To examine the effects of protease inhibitors on the growth and development of *E. integriceps* nymphs an artificial diet was established. Artificial diets were prepared using wheat germ (25%), wheat flour (19%), sucrose (3.5%), yeast extract (7.5%), wheat starch (31.5%), casein (12.5%), wheat germ oil (3.5%) (v/w), and water (44%) (v/w). Pieces of diet similar to wheat grains were prepared and placed on an expanded sheet of parafilm that were wrapped around the diet in a sausage-like shape.

The levels of inhibitor used in the diet were expressed as percentage of protein of inhibitor per protein of diet (W/W). The inhibitors (lyophilized powder) added to the diet were SBTI, TLCK, and TPCK each at two concentrations including 1% and 4% of dietary protein. Also, one more treatment, a combination of TPCK and TLCK (4% each), was used. The control diet was without inhibitors.

Because first instars of *E .integriceps* do not feed and second instar nymphs are small, a third nymphal instar was transferred to the diet with a fine brush and its growth and development were monitored to 24 hours post-emergence of adult. In each treatment (each dose), 12 newly molted third instar nymphs were used and each treatment had five replicates. Diets were replaced twice a week. The insects were kept at 25±2° C and 14:10 (L:D) photoperiod. Nymphal development times, survival rate, and adult weights 24 hours post molt were monitored.

### Preparation of luminal enzyme extract

Enzyme samples from midguts of adults were prepared by the method of Lam et al. (1999) with slight modifications. Briefly, adults were randomly selected and the midgut from these individuals were removed by dissection under a light microscope in ice-cold 50mM Tris-HCl buffer (pH8.0) containing 0.01M CaCl_2_. The midgut contents exuded into the buffer while stirring on ice, and the exudates were centrifuged at 12000g for 10 min at 4° C. The supernatant were pooled (as an enzyme source) and stored at -20° C for subsequent analysis.

### Effect of proteinase inhibitors on endogenous proteolytic enzyme activity

Proteolytic activity in gut from adults fed on SBTI-, TLCK-, and TPCK-containing diets during their nymphal growth and their respective controls were measured using azocasein (general substrate), BApNA (trypsin Substrate), and SAAPFpNA (chymotrypsin substrate). These adults from third nymphal instar to 24 hours post-emergence were fed on diets containing different levels of SBTI (1% and 4% of dietary proteins), TLCK (1% and 4% of dietary proteins), TPCK (1% and 4% of dietary proteins), and a combination of 4% SBTI and 4% TPCK.

### Protease assay using general substrate

General proteolysis was done according to the methods of Elpedina et al. (2001) and Gatehouse et al. (1999) using 2% azocasein as substrate with some modifications. The buffer used was Tris-HCl buffer pH 8.0. The reaction mixture consisted of enzyme (midgut extract), substrate, and Tris-HCl buffer. The reaction started with an addition of substrate, and incubation was done at 30° C. 500 *µl* of reaction mixture were removed at 0, 5, 10, 15, 20, and 30 minutes time intervals and equal amounts of 30% (w/v) TCA was added to each in order to terminate the reaction. The samples were mixed, left to stand for 30 min at 4° C, and centrifuged at 15000 g for 15 min. Supernatant was removed and an equal volume of 1 M NaOH was added to it and absorbance was recorded at 410 nm. Appropriate blanks (no substrate and no enzyme) were run for all assays.

### Protease assay using specific substrate

Trypsin- and chymotrypsin-like activities were assayed based on the methods of Gatehouse et al. (1999) and Elpidina et al. (2001) with slight modifications.

The assay was carried out using 1mM BApNA (Benzoyl-l-arginine-p-nitroanilide) and 1 mM SAAPFpNA (Succinyl-l-Ala-l-Ala-l-Pro-Phe-p-nitroanilide). Substrate was dissolved in dimethyl-sulfoxide (DMSO) and then diluted in buffer and the final concentration of DMSO in solution was less than 10%. The hydrolysis of substrates was monitored continuously at 410 nm at 30° C, and initial rates were measured from the slopes of absorbance against time.

### Proteinase assay using gelatin/PAGE

*Eurygaster integriceps* luminal proteolysis was qualitatively assayed using gelatin/PAGE based on Laemmli (1970) and Walker et al. (1998). Gut extract (2.7 mg/ml of total soluble protein) was run on a 12.5% resolving gel copolymerised with 0.1% gelatin. The sampleloading buffer did not contain mercaptoethanol and samples were not boiled prior to loading. Electrophoresis was conducted at 4° C and then SDS was eluted from the gel by incubation in 2% (v/v) Triton X-100 for 30 min at 37° C. Then, the gel was incubated in Ttris-buffer solution (pH 8) at 37° C for 12 h prior to staining with 40% methanol, 7% glacial acetic acid, and 0.05% coomassie Brilliant Blue R and destained until proteolytic activity was seen as clear bands in a dark background.

### Protein determination

Protein concentration was measured according to the method of Bradford (1976), using bovine serum albumin (Bio-Rad, www.biorad.com) as a standard.

### Statistical Analysis

One-way analysis of variance (ANOVA) was done to compare the data followed by Tukey's test when significant differences were found (SAS Institute 1997).

## Results

**Table 1.**
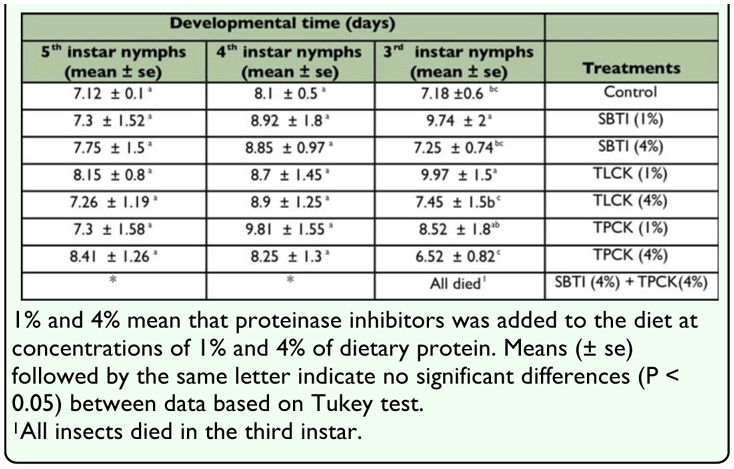
The effects of proteinase inhibitors incorporated into diet on the Sunn pest nymphal development time.

### Effect of inhibitors on development time and survival

Analysis of variance (ANOVA) indicated that protease inhibitors affected nymphal development to some extent (Table 1). Mean development time of third instar nymphs in control, SBTI (1%), TLCK (1%), and TPCK, was 7.18, 9.74, 9.97, and 8.52 days, respectively. Mean development time of third instar nymphs in SBTI (1%) and TLCK (1%) treatments were retarded 2.55 and 2.78 days, respectively.

There were significant differences in third instar mean development time between control and SBTI and TLCK when these inhibitors were present in the diet in 1% concentrations (P < 0.05). Mean development time of the third instar nymphs between SBTI (1%) and TLCK (1%) was not significantly different (P > 0.05).

With incorporation of TPCK into the diet at two concentrations of 1% and 4% of dietary protein, significant retardation in the mean development time of third instar nymphs was not observed. However, development time of third instar nymph for control, 1% TPCK, and 4% TPCK was 7.18, 8.52, and 6.52 days, respectively.

**Figure 1.**
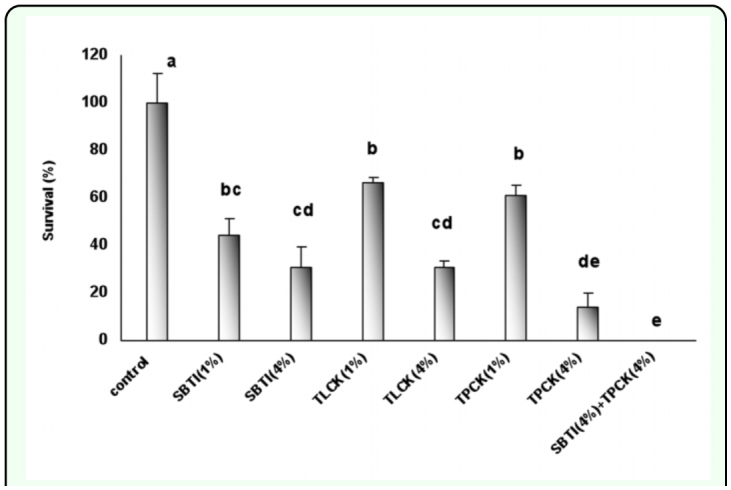
The effect of proteinase inhibitors incorporated into diet on *Eurygaster integriceps* survival. Proteinase inhibitors incorporated into the diet and offered to the third instar nymphs and their survival was measured at 24-hour post emergence of adult. 1% and 4% means that proteinase inhibitors were added to the diet at concentrations of 1% and 4% of dietary protein. Means (± SE) followed by the same letter above bar indicate no significant differences (p < 0.05) between data based on Tukey test. High quality figures are available online.

Using combination of SBTI and TPCK in diets simultaneously caused third instar nymphs to die before reaching fourth instar nymph stage (Table 1, Figure 1). The highest mortality (100 % mortality) observed when combinations of TPCK and SBTI both at 4% of dietary protein were used followed by TPCK (4%) that produced 95% mortality. With increasing concentrations of protease inhibitors, percentage of mortality increased, i.e. SBTI (4%), TLCK (4%), and TPCK (4%) caused 80, 80, and 95% mortality, respectively. There was not a significant difference between mortality in SBTI (4%) and TLCK (4%). However, there was significant difference between TLCK (4%) and TPCK (4%) that produced 80 and 95% mortality, respectively (Figure 1). When low doses of protease inhibitors (1% of dietary proteins) used insect mortality was lower in comparison with high doses of inhibitors. There were significant differences in mortality between low doses of inhibitors and control (P < 0.05). The insect mortality in different inhibitors, when low doses were used, was not significant.

**Figure 2.**
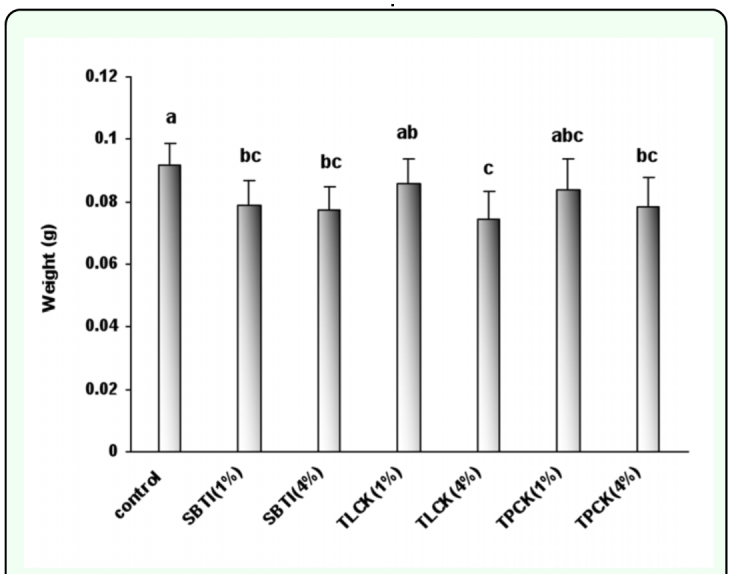
The effect of proteinase inhibitors incorporated into diet on *Eurygaster integriceps* weight. Proteinase inhibitors incorporated into the diet and offered to the third instar nymphs and adult weight was recorded 24-hour post emergence. 1% and 4% means that proteinase inhibitors were added to the diet at concentrations of 1% and 4% of dietary protein. Means (± SE) followed by the same letter above bar indicate no significant differences (p < 0.05) between data based on Tukey test. High quality figures are available online.

### Effect of inhibitors on the Sunn pest weight

The inclusion of the serine protease inhibitors SBTI (1% and 4%), TLCK (1% and 4%), and TPCK (1% and 4%) in diets resulted in reduction of weight when compared with that of control fed insects (P ≤ 0.05) (Figure 2). Insect weight when fed on TPCK (1%) was not significantly different with control; however, the other treatments produced significant differences with control (P < 0.05). Adult weight in control was more than 90 mg; whilst adult weight in SBTI treatments (both 1% and 4% doses), TLCK (4%), and TPCK (4%) was less than 80 mgs.

There were no significant differences in weights among treatments at both 1 and 4% doses (Figure 2).

### Effect of inhibitors on midgut proteinase activity using azocasein

**Figure 3.**
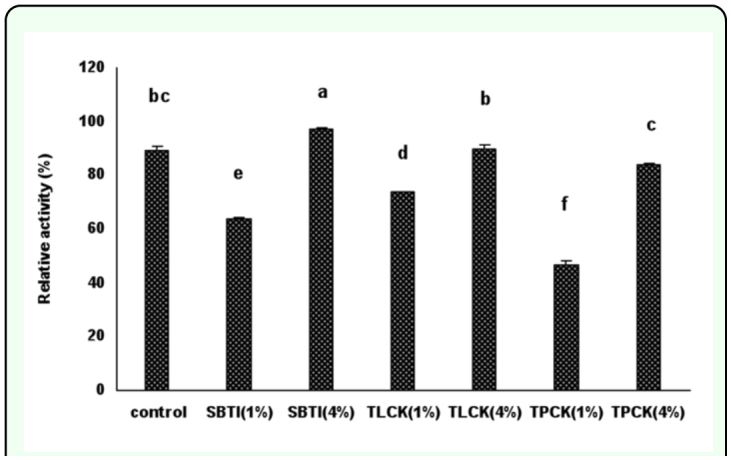
The effect of proteinase inhibitors incorporated into diet on *Eurygaster integriceps* endogenous proteolytic activity using azocasein as a substrate. Proteinase inhibitors incorporated into the diet and offered to the third instar nymphs and adult proteinase was extracted 24-hour post emergence and used as the enzyme source. 1% and 4% means that proteinase inhibitors were added to the diet at concentrations of 1% and 4% of dietary protein. Means (± SE) followed by the same letter above bar indicate no significant differences (p < 0.05) between data based on Tukey test. High quality figures are available online.

Azocaseinolytic activity in gut extract of *E. integriceps* control-fed adults and inhibitorfed adults was examined (Figure 3). General protease activity in protease inhibitor fed adults was different. There were not significant differences in general protease activity between control and TLCK (4%) and TPCK (4%). General protease activity of SBTI-fed adults (4%) was high. However, protease activity in low dosage (1%) inhibitor fed-adults was significantly reduced (P < 0.01). Low doses of SBTI (1%), TLCK (1%), and TPCK (1%) produced more inhibition of protease activity of adults than those of high doses (Figure 3). For example, reduction of protease activity in adult *E. integriceps* when fed on diets containing SBTI (1%), TLCK (1%), TPCK (1%), and TPCK(4%) was 29, 17 ,48, and 6%, respectively.

### Effect of inhibitors on midgut proteinase activity using BApNA substrate

**Figure 4.**
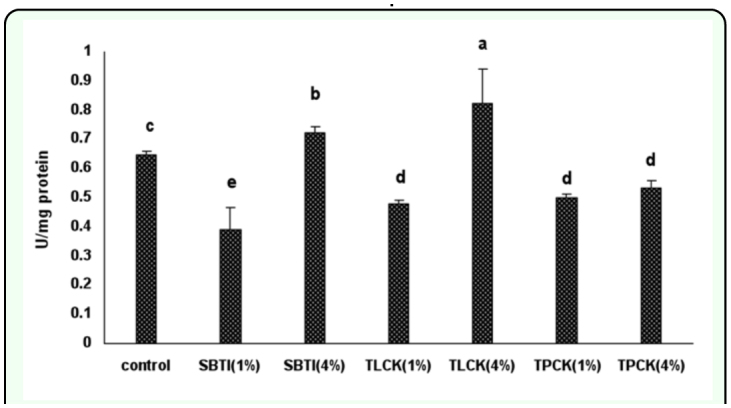
The effect of proteinase inhibitors incorporated into diet on the *Eurygaster integriceps* endogenous proteolytic activity using BApNA, specific trypsin substrate. Proteinase inhibitors incorporated into the diet and offered to the third instar nymph and adult proteinase was extracted at 24-hour post emergence and used as enzyme source. 1% and 4% means that proteinase inhibitors were added to the diet at concentrations of 1% and 4% of dietary protein. Means (± SE) followed by the same letter above bar indicate no significant differences (p < 0.05) between data based on Tukey test. High quality figures are available online.

There were significant differences in protease activity between all treatments and controls when BApNA used as a substrate (P < 0.05) (Figure 4). Trypsin activity significantly reduced in *E. integriceps* guts fed with low doses of SBTI (1%), TLCK (1%), and both doses of TPCK (1% and 4%) in comparison with those of control and reduction was 40, 26, 23, and 17%, respectively.

Reduction of trypsin activity in SBTI was greater than TLCK (1%) and both doses of TPCK (1% and 4%). Trypsin activity in *E. integriceps* guts fed on diets containing high doses of SBTI (4%) was significantly more than those fed on low doses of SBTI (1%), and the same trend was observed regarding TLCK doses. For example trypsin activity in *E. integriceps* guts fed with high doses of SBTI (4%) and TLCK (4%) increased 12 and 27%, respectively (Figure 4).

### Effect of inhibitors on midgut proteinase activity using SAAPFpNA substrate

**Figure 5.**
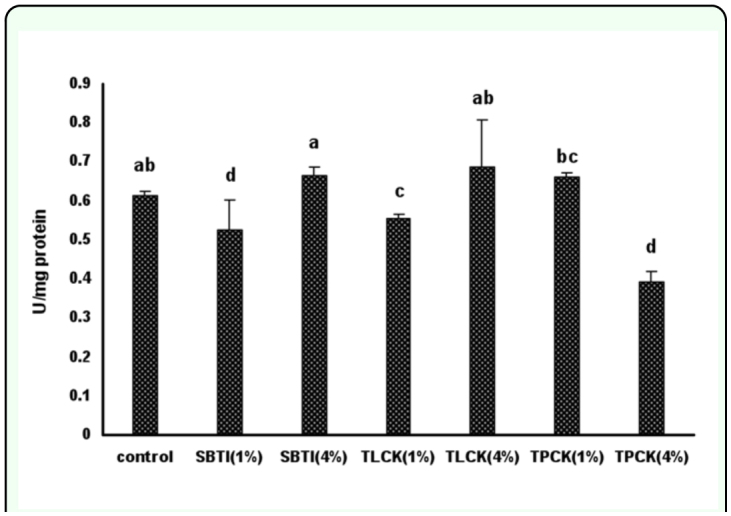
The effect of proteinase inhibitors incorporated into diet on *Eurygaster integriceps* endogenous proteolytic activity using SAAPFpNA, specific chymotrypsin substrate. Proteinase inhibitors incorporated into the diet and offered to the third instar nymphs, and adult proteinase was extracted at 24-hour post emergence and used as enzyme source. 1% and 4% means that proteinase inhibitors were added to the diet at concentrations of 1% and 4% of dietary protein. Means (± SE) followed by the same letter above bar indicate no significant differences (p < 0.05) between data based on Tukey test. High quality figures are available online.

There were significant differences in chymotrypsin activity in adult *E. integriceps* fed on diets containing different concentrations of protease inhibitors (Figure 5). Inhibition of chymotrypsin activity was seen in *E. integriceps* guts fed on SBTI (1%), TLCK (1%), and TPCK (4%) such that inhibition was 14, 9, and 36%, respectively. Maximum inhibition of chymotrypsin activity was observed in *E. integriceps* guts fed on diets containing high doses of TPCK (4%). There were no significant differences observed between high doses of SBTI (4%) and TLCK (4%). However, differences in proteinase activity were seen between *E. integriceps* fed on SBTI (4%) and TLCK (4%) and *E. integriceps* fed on TPCK (4%).

### Effect of inhibitors on midgut proteinase activity using gelatin/PAGE

**Figure 6.**
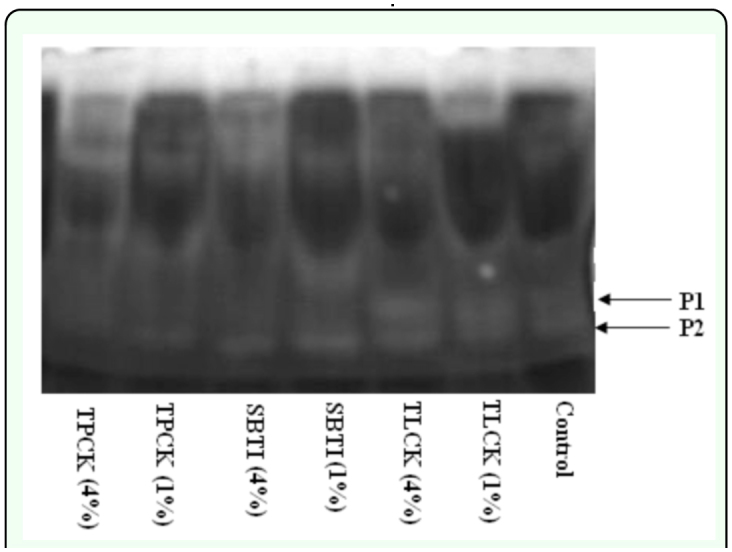
Luminal proteolysis of *Eurygaster integriceps* using gelatin/PAGE. Proteinase inhibitors incorporated into the diet and offered to the third instar nymph and adult proteinase at 24-hour post emergence was extracted and used as enzyme source. 1% and 4% mean that proteinase inhibitors were added to the diet at concentrations of 1% and 4% of dietary protein. The enzyme source (2.7 mg/ml of total soluble protein) was run on a 12.5% resolving gel co-polymerised with 0.1% gelatin. The sample-loading buffer did not contain mercaptoethanol and samples were not boiled prior to loading. Electrophoresis was conducted at 4° C and following electrophoresis SDS was eluted from the gel by incubation in 2% (v/v) Triton X-100 for 30 min at 37° C. The gel was then incubated in Tris-buffer solution (pH 8) at 37° C for 12 h prior to staining with 40% methanol, 7% glacial acetic acid and 0.05% coomassie brilliant blue R and destained until proteolytic activity was seen as clear bands in a dark background. High quality figures are available online.

In gel assays, at least two clear proteinase bands (P1 and P2) were seen in control, i.e. *E. integriceps* fed on diets containing no proteinase inhibitors (Figure 6). In *E. integriceps* fed on proteinase inhibitors, changes were observed in these two bands. When *E. integriceps* fed on diets containing two concentrations of TLCK, these two bands were stronger than the control. However, the Pl band was almost disappeared in *E. integriceps* fed on SBTIs and TPCKs. In addition to P1, the P2 band in *E. integriceps* fed on TPCKs was less intense and almost disappeared compared to *E. integriceps* fed on diets containing 4% of dietary protein TPCK.

## Discussion

In this study, it was found that serine protease inhibitors like SBTI, TLCK, TPCK, and a combination of SBTI and TPCK when incorporated in *E. integriceps* diet at concentrations of 1% and 4% of dietary protein caused significant adverse effects on growth and development as well as gut proteinases.

The effect of individual inhibitors on insect growth and development was not acute and not dose dependent. For example, mean development time of third instar nymphs in 1% SBTI and 1% TLCK treatments were retarded by 2.55 and 2.78 days, respectively. However, when high doses (4%) of SBTI and TLCK were used development retardation was not significant (P > 0.05). Mean development time of the third nymphal stage for control, 4% concentrations of SBTI and TLCK was 7.18, 7.25, and 7.45 days, respectively.

These findings were in accordance with the findings of previous research (Johnston et al. 1993; Gatehouse et al. 1999; Oppert et al. 2003; Silva et al. 2006). For example Gatehouse et al. (1999) found that incorporation of SKTI into the diet at concentration of 2% dietary protein caused development retardation of *Lacanobia oleracea* larvae by 2 days.

Interestingly, retardation of development time was observed only in the third instar nymphs. However, fourth and fifth instar nymphs were not affected significantly by the presence of proteinase inhibitors in their diet (Table 1). It seems that as the nymphs grow and make progress in their development, they adapt to the presence of inhibitors in their diet through a number of mechanisms. These mechanisms, which have been reported in the other insect species, are the stimulation of proteinase activity and increased production of inhibitor insensitive enzymes (Oppert 2000; Oppert et al. 2005; Lawrence and Koundal 2002).

Enzyme assessment showed increments of enzyme activity in the *E. integriceps* when the insect faced high doses of inhibitors in their diet (Figures 3, 4, 5). When the insects fed and grew on low doses of proteinase inhibitors, their enzyme activity was lower than those feeding on high doses of proteinase inhibitors, i.e. feeding on 4% SBTI concentration caused increment of protease activity even more than control insects (Figure 3).

Oppert et al. (2005) showed that additions of cystein proteinase inhibitors in the diets of *Tribolium castaneum* produced a dramatic shift in the insect digestive proteinases from cystein proteinase to serine proteinase.

Adult biomass was also affected by presence of inhibitors in their diet. However, protease inhibitors effects on adult biomass were different. SBTI was the strongest inhibitor in reduction of insect biomass followed by TLCK and TPCK; the two latter inhibitors showed similar results, i.e. their high doses (when incorporated at 4% of dietary protein) caused significant reduction of the insect weight. Johnston et al. (1993) reported that in feeding trials SBTI reduced *Helicoverpa armigera* mean larval weight significantly in comparison with SBBI, Soybean-Bowman-Birk trypsin-chymotrypsin inhibitor. The presence of protease inhibitors in *Tribolium castaneum* diets caused 12–91% reduction in the insect growth (Oppert et al. 2003). Also incorporation of SBTI at concentrations of 3% in *Ceratias capitata* diets caused reduction of larval mass (Silva et al. 2006).

*E. integriceps* survival was affected significantly by the presence of protease inhibitors in their diets. Using a combination of SBTI and TPCK each at 4% concentration of dietary protein caused 100% death of the third instar before reaching the fourth instar suggesting that trypsin and chymotrypsin are important proteases for protein digestion. Each inhibitor individually and dose-dependently caused high percentage mortality, but did not produce 100% mortality indicating that *E. integriceps* could complete its growth with one of the proteases. Although acidic proteases have been shown to be active in *E. integriceps* midgut (unpublished data), alkaline proteases are also vital. It has been reported that in *E. integriceps* salivary glands serine proteinases including trypsin and chymotrypsin are active, but that chymotrypsin is the major proteinase (Hosseini-Naveh et al. 2009). In the current experiment when TPCK (specific inhibitor of chymotrypsin activity) was incorporated into the diet, the lowest activity of general proteases was found indicating that chymotrypsin is the major part of protease activity. Also, the insect survival was affected most by the presence of TPCK in the diet. The lowest survival (less than 20%) of the *E. integriceps* observed in *E. integriceps* fed on a diet containing 4% TPCK. The same trend was seen in the gel assay using gelatin as a substrate. The number of bands visualized in high doses of TPCK was less than the other proteinase inhibitors used. The P1 band disappeared when *E. integriceps* was fed on TPCK and SBTI, and P2 bands almost completely disappeared in the gel prepared from luminal extract of *E. integriceps* fed on TPCK (4%).

*E. integriceps*, as well as the other heteropterans, use macerate (or lacerate) and flush feeding, during which it injects saliva produced in the salivary glands with its piercing-sucking mouthparts into the grain to liquefy the food. The liquefied food is ingested, and further digestion and absorption take place inside the gut (Cohen 2000; Hori 2000; Wheeler 2001; Boyed et al. 2002). The enzymes, especially proteases that are injected into the grain during feeding, degrade high molecular weight subunits of glutenin protein and this results in the production of bread with poor volume and texture (Sivri et al. 2002; Every 1992, 1993; Every et al. 1998; Every and Stufkens 1999; Kinaci and Kinaci 2004; Every et al. 2005). Therefore, blocking activities of the salivary glands' proteolytic enzymes using transgenitic plants could be an important step toward insect control.

In conclusion it should be stated that serine proteinase inhibitors, especially the combination of two inhibitors, have potential for use in Sunn pest, *E. integriceps*, management strategies.
